# Back to the Future: Reviving Old Concepts to Advance Transcatheter Treatment of Calcific Mitral Valve Disease

**DOI:** 10.1016/j.jscai.2025.102638

**Published:** 2025-03-14

**Authors:** Lior Lupu, Toby Rogers, Dan Haberman

**Affiliations:** a Section of Interventional Cardiology, MedStar Washington Hospital Center, Washington, DC; b Georgetown University School of Medicine, Washington, DC; c Cardiovascular Intervention Program, Division of Intramural Research, National Heart, Lung, and Blood Institute, National Institutes of Health, Bethesda, Maryland; d Heart Center, Kaplan Medical Center, Rehovot. Affiliated to the Hebrew University, Jerusalem, Israel

Mitral annular calcification (MAC) is a common degenerative condition, affecting ∼10% of individuals aged over 60 years.^1^ When MAC causes significant mitral valve dysfunction (stenosis, regurgitation, or mixed), both surgical and transcatheter interventions are associated with poor outcomes.^1^ Surgical approaches include mitral valve repair or replacement, with or without extensive calcium debridement. As this is predominantly a disease of old age, many patients are high risk for surgery. Hybrid transatrial mitral valve replacement using a balloon-expandable transcatheter heart valve implanted under direct visualization simplifies valve anchoring but is still surgery. Transcatheter approaches are under investigation, but no US Food and Drug Administration approved transcatheter therapy currently exists. The SAPIEN 3 valve (Edwards Lifesciences) was evaluated in the MITRAL trial for transcatheter mitral valve replacement (TMVR), showing symptom improvement but high 30-day and 1-year mortality rates.^2^ The MITRAL II trial (NCT04408430) of SAPIEN 3-in-MAC has not yet reported its findings but importantly included a natural history cohort that will inform future treatment strategies. Dedicated TMVR devices remain investigational in the setting of MAC-related mitral stenosis (MS).^3^^,^^4^

Percutaneous mitral balloon valvuloplasty (PMBV) is an established therapy for rheumatic MS, effectively restoring valve function by separating fused commissures. Its use in MAC-related MS is limited due to commissural calcification, which increases the risk of leaflet tearing, rupture, and severe mitral regurgitation (MR). Additionally, MAC-related MS often has spicules of annular calcium encroaching onto the leaflets or a bar of calcium across the anterior leaflet, which restrict leaflet mobility. While PBMV may offer some benefit, its outcomes in this setting remain uncertain.^5^

The challenge of heavily calcified mitral valves led to the hypothesis that ultrasound-assisted calcium modification could improve mitral valve interventions. This concept was explored nearly 35 years ago by cardiac surgeons using ultrasonic debridement during mitral valve reconstruction.^6^ Despite its initial promise, the technique was eventually abandoned due to the availability of reliable valve prostheses. With the availability of transcatheter intravascular lithotripsy (IVL) balloons, this concept has resurfaced. IVL integrates ultrasound lithotripsy within a semicompliant balloon (Shockwave Medical) equipped with multiple emitters that generate sonic pressure waves to selectively fracture calcium within a vessel wall. Case reports and small case series have described its use in patients with MAC before PBMV and before TMVR.

In this issue of *JSCAI,* El-Sabawi et al^7^ summarize this published literature in a systemic review.^7^ In the control group, 44 patients underwent conventional PMBV, which demonstrated variable success, limited mitral gradient reduction, and high rates of MR (9.1%) and reintervention (13.6%). In contrast, in the intervention group, lithotripsy (n = 40) resulted in greater mean gradient reduction (5-8 mm Hg) and lower incidence of MR (2.5%) and reintervention (2.5%). The authors concluded that lithotripsy-facilitated PMBV is a feasible approach for MAC-related MS and may provide superior short-term outcomes compared with conventional PMBV, while emphasizing the need for larger studies to evaluate its efficacy and safety.

Several issues must be highlighted when interpreting these findings. While most patients had MAC-related MS, the cohort also included rheumatic cases, where PBMV is expected to be more effective. Patients with varying degrees of MR were included, further contributing to heterogeneity. Moreover, while PBMV was the sole treatment in the control group, the lithotripsy group included patients who received lithotripsy alone or as a pretreatment for PBMV, TMVR, or TEER, which are not comparable treatments. The number and sizes of off-label peripheral lithotripsy balloons (7.0-12.0 mm) used varied between patients. Multiple balloons were sometimes used simultaneously with the hypothesis that this would improve contact with the calcified tissue. The absence of large balloons capable of filling the mitral orifice, combined with the heterogeneous calcium distribution in MAC-related MS, raises concerns about whether sufficient and consistent contact with the calcified tissue really can be achieved to ensure effective calcium modification. Furthermore, the included literature consisted solely of case reports and case series, which are inherently subject to publication bias favoring positive outcomes. Thus, these findings should be interpreted with caution. This pooled data analysis lays the groundwork for a dedicated prospective trial to evaluate the role of lithotripsy and PBMV in MAC-related MS. Dedicated large IVL balloons for valve interventions are in development, with aortic stenosis as the likely first target due to its prevalence and the availability of effective transcatheter therapies. Future research may explore their role in MAC-related MS.

TMVR theoretically provides the most comprehensive solution by addressing both stenosis and regurgitation. In this context, predeployment calcium modification may improve valve expansion, apposition, and paravalvular leak—potential benefits that may extend to SAPIEN 3-in-MAC interventions. Interim results from the ongoing SUMMIT trial, presented at TCT 2023, included 103 patients with MAC who underwent transapical implantation of the Tendyne valve (Abbott). These early findings demonstrated promising outcomes, with a technical success rate of 94% and a 30-day survival rate of 93%, along with the elimination of MR and a reduction in transvalvular gradient. No data were provided about pre-TMVR balloon usage. The main reasons for exclusion were left ventricular outflow tract obstruction risk, out-of-range annular dimensions, and fear for calcium interaction with the inner valve frame. Another dedicated device under investigation is the AltaValve (4C Medical Technologies), which has a spherical frame to fill the left atrium for anchoring. The actual valve sits just above/inside the mitral annulus to reduce the footprint in the left ventricular outflow tract. Initially intended for patients with MR, this valve is currently being tested in a cohort with MAC-related mitral stenosis. If TMVR proves to be an effective treatment for MAC-related MS, PMBV and lithotripsy-facilitated PMBV will likely have a limited role as stand-alone therapies but may still be used to prepare the landing zone for these implants.

In conclusion, MAC-related MS remains a challenging condition with limited treatment options. In general, PBMV is not recommended due to high risk of mechanical complications. Published outcomes after SAPIEN 3-in-MAC are poor in this patient population, and dedicated TMVR devices are still being evaluated. It is theoretically possible that lithotripsy as an adjunctive therapy may enhance the efficacy of PBMV while reducing complications, but this can only be determined in a prospective randomized study and ideally with dedicated larger IVL balloons. In the absence of such data, this concept remains unproven.
